# Microtubule inner proteins in apicomplexan parasites

**DOI:** 10.1042/BST20253110

**Published:** 2026-02-02

**Authors:** Annika M. Binder, Friedrich Frischknecht, Franziska Hentzschel

**Affiliations:** 1 Integrative Parasitology, Centre for Infectious Diseases, Heidelberg University Medical Faculty, Heidelberg, 69120, Germany; 2 German Center for Infection Research, DZIF, Partner site Heidelberg, Heidelberg, Germany

**Keywords:** Apicomplexa, Microtubules, microtubule inner proteins, *Plasmodium*, *Toxoplasma*

## Abstract

Microtubule inner proteins (MIPs) are integral components within the microtubule lumen of various organisms, contributing to microtubule structural integrity and functionality. Apicomplexan parasites, including *Plasmodium spp*. and *Toxoplasma gondii*, exhibit a range of specialized tubulin structures, such as axonemal microtubules, subpellicular microtubules (SPMTs), and conoid fibers, playing critical roles in cellular morphology and motility. Yet, in contrast with model organisms, only a few MIPs have been characterized in apicomplexans so far. Recent advances in cryo-electron tomography and structural proteomics have facilitated the study of MIPs, shedding light on unique adaptations that distinguish apicomplexan microtubules from those in model eukaryotes. Key findings include the identification of an interrupted luminal helix in SPMTs, which is critical for stabilizing microtubules under stress. The relatively small repertoire of axonemal MIPs contrasts markedly with the numerous MIPs observed in other systems, possibly reflecting adaptations for rapid microtubule assembly without intraflagellar transport. Furthermore, emerging evidence points to multiple MIPs within the conoid and SPMTs, suggesting further roles for MIPs in these parasites. This review highlights the currently known contributions of MIPs to the survival and proliferation of these parasites, while emphasizing the need for continued research to fully characterize their diverse roles and molecular mechanisms.

## Microtubules are essential components of the cytoskeleton

**Figure 1:**
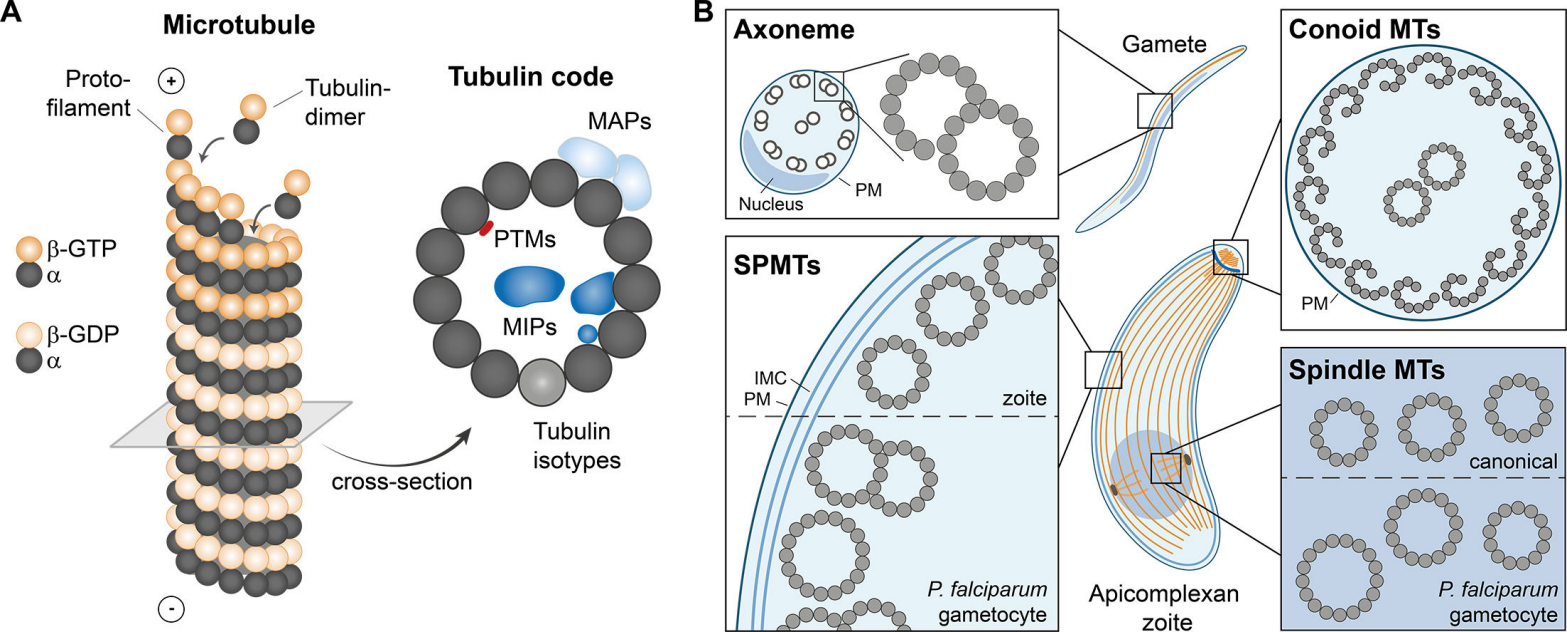
Canonical microtubule and microtubule arrangements found in apicomplexans. **(A**) Assembly of a canonical microtubule formed of 13 protofilaments. A ‘tubulin code’ comprised of microtubule-associated proteins (MAPs), including microtubule inner proteins (MIPs), post-translational modifications (PTMs), and tubulin isotypes determines microtubule properties such as stability and flexibility. (**B**) Microtubule and tubulin arrangements found in apicomplexan gametes, motile stages (zoites), and *Plasmodium* sexual stages (gametocytes). MTs, microtubules, SPMTs, subpellicular microtubules, IMC, inner membrane complex, PM, plasma membrane.

Microtubules are highly conserved across eukaryotes and together with actin and intermediate filaments form part of the cytoskeleton. They are hollow filaments composed of α- and β-tubulin heterodimers that bind to each other longitudinally, forming a protofilament. In a canonical microtubule, 13 protofilaments arrange in a circular fashion to form a tubule (Figure 1A). Microtubule polymerization is driven by GTP hydrolysis, and only GTP-bound tubulin dimers can be incorporated into the growing filament. Both α- and β-tubulin can bind GTP, but only β-tubulin possesses an intrinsic GTPase activity and thus can hydrolyze GTP to GDP and phosphate (reviewed in [1,2]). Following incorporation of a GTP-bound heterodimer, a brief delay in GTP hydrolysis results in the formation of a stabilizing GTP cap. This cap promotes microtubule polymerization and supports continued microtubule growth (reviewed in [3]). Microtubules are highly dynamic structures, constantly alternating between phases of growth and shrinkage, a phenomenon known as dynamic instability [4].

Microtubules can assemble into various structural configurations within the cell, including singlet, doublet, and triplet microtubules, as well as more complex arrangements such as a mitotic spindle essential for chromosome segregation and axonemes, which constitute the core of flagella and cilia and typically consist of two inner singlets surrounded by nine outer doublets. Additionally, cytoplasmic microtubules contribute to the cellular cytoskeleton and thus the wide range of cellular shapes observed across different organisms, while also providing tracks for intracellular transport of cargo.

The dynamic behavior of microtubules is fine-tuned by several factors that govern their stability, function, and identity. Collectively known as the tubulin code [5], these factors include the expression of distinct α- and β-tubulin isotypes, post-translational modifications, and interactions with microtubule-associated proteins (MAPs) (reviewed in [6]) (Figure 1A). While MAPs universally bind to the microtubule lattice, their functions vary significantly. Some MAPs stabilize microtubules and promote polymerization, while others act as destabilizers, shifting the equilibrium toward free tubulin heterodimers. Nucleators, such as the well-characterized γ-tubulin ring complex (reviewed in [7]), initiate microtubule assembly, whereas motor proteins like kinesins and dyneins facilitate intracellular transport by moving cargos along the microtubule network (reviewed in [8,9]).

The first evidence that a subset of MAPs associates uniquely with the microtubule lumen came from electron microscopy of microtubules in frogs [10]. Yet only when more advanced cryo-electron tomography (cryo-ET) enabled scientists to visualize native cellular structures, including microtubules, in high resolution, could these microtubule inner proteins (MIPs) be resolved [11–14] and eventually identified [15,16]. Now, multiple studies revealed an abundance of MIPs, in particular in the lumen of axonemal microtubules of metazoan cilia and flagella, as well as in the flagella of protozoans [17–21]. In apicomplexan parasites, however, only a limited number of MIPs have been characterized to date. In this review, we summarize the MIPs currently identified in apicomplexans and explore their roles in parasite development and infectivity.

## Diverse tubulin structures are found in apicomplexans

Apicomplexan parasites are a diverse group of protozoan pathogens responsible for significant morbidity and mortality in both humans and animals, including livestock. This phylum includes *Plasmodium* spp., the causative agents of malaria; *Toxoplasma gondii* (*T. gondii*), which infects nearly one-third of the global population; and other medically and agriculturally relevant genera such as *Cryptosporidium*, *Theileria*, and *Eimeria*. Within these parasites, a variety of specialized microtubule structures play crucial roles in their cellular organization, replication, and infectivity (reviewed in [22]) (Figure 1B).

Unlike free-living protozoans that rely on flagellar motility, apicomplexan parasites primarily utilize an actin-myosin-based gliding mechanism for movement. As a result, flagellated stages are only present in some parasites during a brief part of their life cycle in male gametes. In malaria parasites, gametes move by an **axoneme** exhibiting the classical 9 + 2 microtubule arrangement [23]. Notably, *Plasmodium* spp. lack the intraflagellar transport machinery typically required for axoneme assembly at the plasma membrane and instead assemble their axonemes within the cytoplasm [24]. The *Plasmodium* axoneme originates from a basal body consisting of 9 singlet A-tubules, which is part of the bipartite microtubule-organizing center (MTOC) embedded in the nuclear envelope [23,25,26]. This MTOC also nucleates spindle microtubules within the nucleus [23,27]. Little is known about how the basal body is built up in *Toxoplasma*, as gametes form only in the intestine of cat hosts and are thus not easily experimentally accessible [25]. Interestingly, male gametes of *Cryptosporidium* spp. lack flagella and instead feature a distinct cage-like microtubule structure surrounding the nucleus [28].

Within the apicomplexans, coccidian parasites possess a **conoid**, a specialized apical structure [29]. In *T. gondii* and the close relative *Neospora caninum*, the conoid consists of tubulin-based fibers that show a unique, comma-shaped arrangement of nine protofilaments. Fourteen of these conoid fibers assemble into a barrel-like structure, with two **intraconoidal microtubules** extending through its center [30–33]. While previously thought to be absent from the Aconoidasida (which includes *Plasmodium* spp.), recent studies have revealed that much of the conoid proteome is conserved across Apicomplexa and homologues of several *T. gondii* conoid proteins localize to the apical region of *Plasmodium*, suggesting the presence of a functional equivalent in these parasites [34–36].

In addition to a conoid, all apicomplexans also possess **subpellicular microtubules (SPMTs**), which are crucial for determining parasite shape and providing intrinsic cell stability. These microtubules are nucleated from the apical polar ring and tightly associated with the inner membrane complex, flattened vesicles directly underlying the plasma membrane [36–38]. SPMTs are highly stable and resistant to depolymerization by classical microtubule-targeting drugs or detergents [39,40]. The precise number and arrangement of SPMTs varies between species and stages, ranging between one and nine in *Plasmodium* merozoites (the stage infecting red blood cells), 22 in *Toxoplasma* tachyzoites (the rapidly dividing, invasive stage responsible for acute infection), and up to 60 in *Plasmodium* ookinetes (the stage invading the mosquito midgut) [34,41]. SPMTs are usually 13-protofilament singlets, and their organization is tightly regulated. However, it was recently shown that *P. falciparum* sexual stages (gametocytes) form SPMTs that can not only consist of up to 18 protofilaments but also arrange in doublets or even triplets [36].

Finally, as other organisms, apicomplexan parasites also depend on **spindle microtubules** during mitosis to enable chromosome segregation. Typically, spindle microtubules are 13-protofilament singlets nucleated from centrosomes containing centrioles. *T. gondii* and other coccidians retained centrioles, but these consist of an unconventional 9 + 1 singlet structure instead of the more canonical arrangement of nine triplet microtubules found in other eukaryotes [25,40]. Many other apicomplexans, including *Plasmodium* spp., lack centrioles altogether [25]. Instead, these species rely on an amorphous, acentriolar MTOC known as centriolar plaque for spindle microtubule nucleation [42]. Notably, *P. falciparum* gametocytes are again exceptional, with much larger spindle microtubules consisting of on average 15 and up to 17 protofilaments [36]. In contrast with the other microtubule structures described, spindle microtubules are highly dynamic, and thus unlikely to be stabilized by associated MIPs.

## Microtubule inner proteins in apicomplexans

Over the past five years, significant progress has been made in using cryo-ET to examine the microtubule lumen in apicomplexan parasites. In addition, the development of structure prediction tools enabled the identification of putative MIP homologs by structure similarity. These studies have revealed the presence of MIPs in multiple microtubule structures in apicomplexans and allowed their functional characterization.

## Few MIPs in axonemal microtubules of apicomplexans

MIPs were initially identified in the lumen of axonemal microtubule doublets [12]. To date, a plethora of MIPs have been shown to be present within the doublet microtubules of *Tetrahymena* cilia, *Chlamydomonas* flagella, and sea urchin sperm [12–14,17–19] but also in human sperm axonemes [19,43,44], and the flagella of various protists [20,21,45]. Within these structures, MIPs are found in both the A- and B-tubules, although the majority localize to the A-tubule. Mammalian sperm cells possess the highest number of known MIPs, with 57 distinct proteins decorating their axonemal doublet microtubules [19], while *Chlamydomonas* axonemes contain 33 MIPs [17].

**Figure 2:**
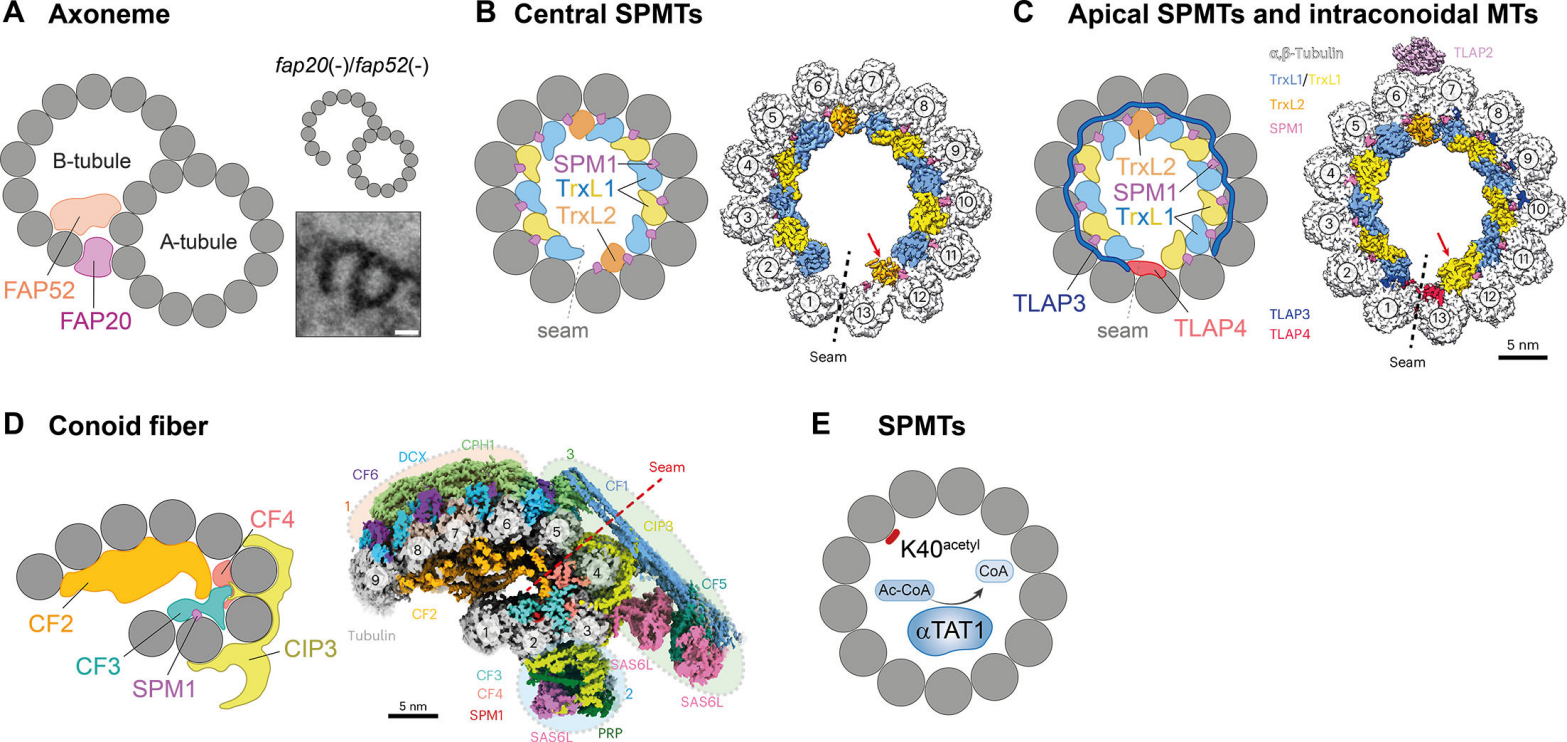
MIPs in apicomplexans. (**A**) Model of axonemes, showing how FAP20 and FAP52 localize to the inner junction of the B- to the A-tubule. Right: In the absence of both proteins, the B-tubule disconnects from the A-tubule. Data reprinted with permission from [46]. (**B**) Model of central SPMTs with MIPs decorating the lumen (SPM1, TrxL1, and TrxL2). Right: Cryo-EM structure of *T. gondii* central SPMTs with SPM1 (pink), TrxL1 (blue/yellow), and TrxL2 (orange) highlighted. **C**) Model of *Toxoplasma* apical SPMTs and intraconoidal microtubules decorated with MIPs (SPM1, TrxL1, TrxL2, TLAP3, and TLAP4). Note that the model is flattened to depict a full TLAP3 molecule wrapping around the inside lumen. Right: Cryo-EM structure of *T. gondii* apical SPMTs with SPM1 (pink), TrxL1 (blue/yellow), TrxL2 (orange), TLAP3 (dark blue), and TLAP4 (red) highlighted. Additionally, the MAP TLAP2 (purple) is depicted [47] Note that protofilament 12 and 13 are occupied by TrxL2 in the central region of SPMTs and by TrxL1 in the apical region of SPMTs (red arrow). (**D**) Model of a conoid fiber with associated MIPs (SPM1, CF2, CF3, and CF4). Right: Cryo-EM structure of *T. gondii* conoid fiber with associated MIPs and MAPs [33]. (**E**) Model of α-TAT1 catalyzing the acetylation of lysine 40 (**K40**) within the lumen of SPMTs using acetyl-coenzyme A (acetyl-CoA) as donor. (**B-D**) Structures adapted from [48].

In apicomplexan parasites, axonemal MIPs have so far been characterized exclusively in the rodent malaria parasite *P. berghei*. Of the multitude of MIPs identified in *Chlamydomonas* axonemes, two were identified in *Plasmodium* based on sequence and structure similarity: flagellar-associated protein 20 (FAP20) and flagellar-associated protein 52 (FAP52) (Figure 2A) [46]. Upon individual knockout, both were found to be dispensable for parasite development in the mammalian and mosquito host, with only a mild reduction in the formation of ookinetes (the motile zygotes of *Plasmodium*) observed in *fap52*-deficient parasites [46]. In contrast, the combined deletion of *fap20* and *fap52* abolished the formation and motility of male gametes, leading to a complete developmental block in the mosquito. Electron tomography of the double knockout showed that the B-tubule periodically detached from the A-tubule [46], supporting a role of these proteins in maintaining doublet microtubule stability and enabling effective force transmission for flagellar beating. The core function of FAP20 and FAP52 is thus conserved across species, as a similar phenotype has been described for *fap20*/*fap52* mutants in *Chlamydomonas* [49]. Homologues of FAP20 and FAP52 are present across apicomplexans. Canonically, FAP20 forms an alternating ladder together with Parkin co-regulated gene (PACRG) at the inner junction, connecting the B with the A tubule [16]. Curiously, sequence- and structure prediction-based homology searches do not yield a homologue of PACRG in *Plasmodium*, while a homologue is readily found in *Toxoplasma* [50,51], It remains to be investigated if an extended domain present in *Plasmodium* FAP20 compensates for the loss of PACRG or if there is a different protein fulfilling the role of PACRG in *Plasmodium*.

The apparent paucity of conserved MIPs in apicomplexan axonemes has recently been challenged by two structural proteomics studies in trypanosomatid parasites, which identified a remarkable number of 154 axonemal-associated proteins, including 52 MIPs in *Trypanosoma brucei* and 51 in *Leishmania tarantolae* and *Crithidia fasciculata* [20,21]. From these MIPs and using a PsiBlast search strategy, the authors identified a conserved set of six proteins as ‘core MIPs,’ present across all organisms examined—including *Plasmodium* [20]. Besides FAP20 and FAP52, these core MIPs include FAP106 (binding near the inner junction), the filamentous MIPs FAP45, FAP53, FAP127, and FAP210 that bind to the cleft between protofilaments, and the more globular FAP115. The function of these putative *Plasmodium* axonemal MIPs remains, however, to be investigated. The relatively low number of MIPs in *Plasmodium* correlates with a general reduction in basal body and flagellar components among apicomplexans [52], and may be attributed to the rapid assembly of axonemes within the cytoplasm, the absence of intraflagellar transport, and the typically short lifespan of gamete flagella. Therefore, *Plasmodium* possibly tolerates reduced axoneme stability and integrity better than trypanosomatid parasites, whose flagella persist for longer periods. It is, however, also possible that yet unidentified, apicomplexan-specific axonemal MIPs take on stabilizing functions, in analogy to the large number of trypanosomatid-specific MIPs now identified [20,21]. Overall, the comparably limited knowledge about apicomplexan axonemal MIPs highlights both the limitations of earlier studies and the need for further functional investigations into the roles of these additional MIPs in *Plasmodium* biology.

### MIPs decorate the lumen of SPMTs, forming an interrupted luminal helix

A first cryo-ET study identified the presence of a periodic protein density in the lumen of *Toxoplasma* and *Plasmodium* SPMTs [53]. Recently, this protein density could be resolved at higher resolution and identified as an interrupted luminal helix composed of multiple MIPs [36,47]. In *Toxoplasma*, this luminal helix consists of subpellicular microtubule protein 1 (SPM1), which links thioredoxin-like protein 1 (TrxL1) and TrxL2 to the microtubule lattice (Figure 2B). Functionally, these proteins appear to mediate interactions between tubulin monomers: SPM1 spans the longitudinal interface between α- and β-tubulin, while TrxL1 and TrxL2 facilitate lateral connections between protofilaments [47]. Together, these three proteins form two half-crescents, each consisting of five copies of TrxL1, six copies of SPM1, and one copy of TrxL2 [47]. These half-crescents are positioned on either side of the microtubule seam, thereby maintaining an interrupted organization [33,47]. This structural arrangement is conserved in *Plasmodium* spp.; however, as *Plasmodium* spp. do not encode TrxL2, there is an unoccupied space at the respective position [36]. Interestingly, this interrupted luminal helix is present only in the motile mosquito stages (ookinetes and sporozoites) of *Plasmodium* but is absent from merozoites and gametocytes [36].

SPM1 is a conserved MIP found almost exclusively in apicomplexan species, including *Plasmodium* spp., *T. gondii*, *N. caninum*, and *Cryptosporidium* spp. The *spm1* genes across species share conserved N- and C-terminal regions, separated by a variable number of internal amino acid repeats [39]. While *T. gondii*, *N. caninum*, and *C. muris* encode five to six repeats within their *spm1* gene, all *Plasmodium* species harbor at least seven, with *P. vivax spm1* containing the highest number of thirteen repeats [39]. In *T. gondii*, truncation analyses demonstrated that a minimum number of four repeats is necessary for proper microtubule localization [39]. Structural comparisons revealed that the protein shares high similarity with FAP363, a ciliary MIP in *Chlamydomonas* [17,47]. Both are filamentous proteins that span the interface between α- and β-tubulin and contain a conserved Mn-motif, a motif that is also present in many other MAPs and is associated with microtubule stabilization [47,54,55].

The thioredoxin-like proteins TrxL1 and TrxL2 are named after their thioredoxin fold—a conserved feature of the thioredoxin family—but they lack the canonical CXXC motif necessary for enzymatic redox activity [56] (reviewed in [57]). TrxL1 is broadly conserved across most apicomplexan parasites, whereas TrxL2 is restricted to coccidian parasites [58]. In *T. gondii*, TrxL1 localizes to both SPMTs and intraconoidal microtubules, whereas TrxL2 was found exclusively associated with SPMTs [56]. Both proteins require SPM1 for microtubule association [47,56]. Co-immunoprecipitation of TrxL1 in *T. gondii* uncovered its interaction with tubulin, SPM1, and four TrxL1-associated proteins (TLAPs) [56]. While TLAP1 and TLAP2 are conserved between *T. gondii* and *Plasmodium* spp., *Tg*TLAP3 shares only weak homology with its *Plasmodium* orthologue, and TLAP4 is restricted to coccidian parasites [56]. All TLAPs exhibit distinct patterns of SPMT localization and were initially proposed to function as outer MAPs [56,58]. However, recently, TLAP3 and TLAP4 were discovered to be MIPs, too, as discussed below [48].

Surprisingly, individual deletion of *spm1*, *trxL1* (in *T. gondii* and *P. berghei*), or *trxL2* (in *T. gondii*) did not affect SPMT formation and parasite fitness under standard conditions [39,46,47], although *spm1*-KO *T. gondii* were outcompeted by wild-type in co-culture assays [39]. Also, double knockouts of both *trxL1* and *trxL2* in *Toxoplasma*, or *spm1* and *trxL1* in *P. berghei,* did not show any defects, highlighting the high intrinsic stability of SPMTs and possibly functional redundancy among SPMT-associated proteins [46,47]. However, this stability can be challenged under suboptimal conditions or stress: In *T. gondii*, exposing single or double MIP knockout mutants (*spm1*-KO, *trxL1*-KO, *trxL1*/*trxL2*-KO) to chemical stress led to a marked reduction in SPMT length, indicating a protective role of these proteins under destabilizing conditions [47]. In a separate approach, the combined deletion of *spm1* and TrxL1-associated proteins *tlap2* and *tlap3* in *T. gondii* led to significantly shorter SPMTs even under normal conditions, resulting in more straight rather than helical parasite motility patterns [59]. These microtubules were further destabilized by cold treatment, leading to almost complete depolymerization [59]. Together, these findings show that while MIPs may be functionally redundant under optimal conditions, the stability of SPMTs under stress conditions relies on the combined contributions of both MIPs and MAPs.

A recent cryo-EM study specifically resolved the *Toxoplasma* conoid and nearby apical microtubules at near atomic resolution, revealing 40 conoid proteins, including new MIPs [48]. One major finding was that the apical section of the SPMTs and the two intraconoidal microtubules contain a more complex MIP arrangement compared with the central parts of SPMTs (Figure 2C). While the central MIP scaffold is still made up of the helix composed of SPM1, TrxL1, and TrxL2, the second copy of TrxL2 is replaced by a copy of TrxL1, possibly explaining why previous studies could not detect TrxL2 at the intraconoidal microtubules [56]. In addition, TLAP3 and TLAP4 localize to the microtubule lumen (Figure 2C). TLAP4 binds to the inside of the microtubule seam. TLAP3 is a so-called arc-MIP, binding laterally along the inside of 11 of the 13 protofilaments. Compared with TLAP3 and TLAP4, TLAP2 was confirmed to be an outer MAP, binding specifically to the surface of apical SPMTs.

### Conoid fibers are highly decorated with MIPs

Conoid fibers have a highly unusual protofilament arrangement, with a pronounced kink between protofilament 3 and 4, yielding the characteristic comma shape. In its lumen, a protein density has been observed, possibly stabilizing this shape, but the corresponding proteins could not be identified until recently [30,33]. Now, the structure of conoid fibers has been resolved by CryoEM, identifying many proteins decorating these microtubules, including four MIPs in the conoid fiber lumen (Figure 2D) [48]. One of them was SPM1, while the other MIPs found in SPMTs were not present in the conoid. In addition, the conoid fiber proteins CF2, CF3, and CF4 were identified as conoidal MIPs (Figure 2D). CF2 forms long coiled-coil bundles binding longitudinally to the protofilaments, while CF3 and CF4 are more globular proteins binding on the inside of the kink of the comma [48]. In addition, the conoid protein hub 1 (CPH1)-interacting protein CIP3 penetrates through the protofilaments, localizing partially to the lumen of the conoid fibers, while the outer part wraps around the protofilaments (Figure 2D) [48,60]. Of these conoid MIPs, only CF4 has a homologue in *Plasmodium*, in line with the highly reduced conoid structure of these parasites [48]. Strikingly, despite its prominent presence in the conoid, deletion of CF2 has no effect on *T. gondii* growth *in vitro* [60]. Likewise, CF4 was shown to be non-essential for *Toxoplasma* tachyzoites, CIP3 deletion only causes a moderate growth defect, and CF2 is predicted to be non-essential according to a genome-wide screen [60,61]. Conoidal MIPs, like SPMT MIPs, may thus be functionally redundant to ensure the high structural stability of the conoid fibers.

### Alpha tubulin acetyltransferase 1 (α-TAT1)

The α-tubulin acetyltransferase 1 (α-TAT1) is an enzyme originally identified in *Caenorhabditis elegans* [62] (reviewed in [63]). Its best-characterized function is the acetylation of lysine 40 of α-tubulin, a residue located on the luminal side of microtubules (Figure 2C). Acetylation of lysine 40 is associated with stable, flexible microtubules, but its modification has only mild phenotypes in a variety of species [63]. In addition to its enzymatic activity, several studies suggest that α-TAT1 also has a structural function in the lumen of microtubules [63]. In apicomplexans, the functional role of α-TAT1 and lysine 40 acetylation was first investigated in *T. gondii*, where lysine 40 acetylation was observed both in subpellicular and spindle microtubules (Figure 2E) [64]. Disruption of *Tg*α-TAT1 led to a loss of lysine 40 acetylation and significantly impaired centrosome duplication, karyokinesis, and apicoplast division, ultimately arresting parasite replication [64]. However, subsequent analysis using different mutants showed that *Tg*α-TAT1 is not essential for *Toxoplasma* tachyzoites [65]. In *Plasmodium*, the enzyme α-TAT1 and the lysine at position 40 of α-tubulin are conserved, with the notable exception of rodent-infecting species [66]. These species lack α-TAT1 and encode glutamine instead of lysine at position 40, mimicking acetylated lysine and possibly providing permanent microtubule stability. However, mutation of the glutamine to lysine in the absence of α-TAT1 did not affect rodent *Plasmodium* life cycle progression under laboratory settings, suggesting a redundant role for acetylation like in *T. gondii* [65].

### Exploring the unknowns

Compared with model eukaryotes, only a few MIPs have been identified in Apicomplexa, and it is likely that additional MIPs are still out there to be discovered. Some have already been described as yet unidentified densities in cryo-ET studies. For example [30,33], in *Toxoplasma* SPMTs, a small, unidentified density was found at the Taxol-binding site of the β-tubulin subunit [47]. While too small to be a protein, it remains to be determined if this density corresponds to a small molecule stabilizing the cortical microtubules. Also, cryo-ET of *Plasmodium* ookinete SPMTs indicates the possible presence of MIPs in the lumen of microtubule minus ends [36]. High-resolution structures of axonemes are currently being generated and might reveal additional MIPs in these microtubules [26].

## Summary

In conclusion, the study of MIPs in apicomplexans has unveiled their critical roles in microtubule stability, motility, and cellular organization, reflecting the unique adaptations of these parasites to their host environment. Despite significant advancements in recent years, the known MIP repertoire in apicomplexans remains small, and it is likely that new MIPs will be discovered and functionally characterized. Future research leveraging technologies such as cryo-ET and structural proteomics promises to deepen our understanding of these proteins, offering new insights into the molecular mechanisms governing parasite survival and proliferation.

**Perspectives**

- Microtubule inner proteins (MIPs) are crucial factors regulating microtubule function, specialization, and dynamics, yet apicomplexan parasites seem to contain a reduced and/or divergent set of MIPs showcasing their evolutionary divergence from model organisms.
- While only a few MIPs have been identified so far in apicomplexans, some of those characterized were found to play vital roles in stabilizing microtubules, especially under stress.
- Advanced techniques like cryo-electron tomography and proteomics are pivotal for identifying new, possibly lineage-specific MIPs, which will shed light on parasite-specific microtubule dynamics.

## Open questions

1. In apicomplexan axonemes, only two MIPs were so far functionally characterized, and four additional MIPs are predicted to be present. Compared with the over 30 MIPs identified in other eukaryotes, how can this limited MIP repertoire provide sufficient stability to the axonemes?
2. In the absence of a clear PACRG homologue in *Plasmodium*, how is the seam between the B- and the A-tubules of axonemes formed in this parasite?
3. Do apicomplexans, like trypanosomes, possess divergent, so far undiscovered lineage-specific MIPs?
4. What is the biological relevance of the unusual protofilament numbers in *P. falciparum* gametocyte SPMTs and spindle microtubules, and how are the SPMT doublets and triplets organized in these parasite stages?

## Abbreviations

CF: conoid fiber
CIP3: conoid protein hub 1-interacting protein 3
CPH1: conoid protein hub 1
FAP: flagellar-associated protein
KO: knockout
MAP: microtubule-associated protein
MIP: microtubule inner protein
MT: microtubule
MTOC: microtubule-organizing center
PACRG: Parkin co-regulated gene
SPMT: subpellicular microtubule
TLAP: TrxL1-associated protein
TrxL: thioredoxin-like protein
cryo-ET: cryo-electron tomography
α-TAT1: α-tubulin acetyltransferase 1
